# Family Engagement in a Digital Intervention Targeting Risk for Anxiety in Parent-Child Dyads: Mixed Methods Study

**DOI:** 10.2196/79898

**Published:** 2026-04-09

**Authors:** Isaac A Mirzadegan, Ericka M Lewis, Sally L Cole, Alexandria M Meyer

**Affiliations:** 1 Department of Psychology, Florida State University, Tallahassee, FL, United States; 2 San Francisco VA Medical Center, 4150 Clement St (116B), San Francisco, CA, 94121, United States, 1 415-221-4810 ext 24953; 3 Department of Psychiatry and Behavioral Sciences, University of California, San Francisco, San Francisco, CA, United States; 4 School of Social Work, University of Maryland, Baltimore, Maryland, United States; 5 School of Education and Counseling Psychology, Santa Clara University, Santa Clara, United States

**Keywords:** anxiety, mobile health, digital health, engagement, parenting intervention, prevention, perfectionism, single-session intervention, cognitive behavioral therapy, preschool, adherence, fidelity, eHealth, mHealth

## Abstract

**Background:**

Digital health programs are increasingly important in the treatment and prevention of mental health problems in young children. However, suboptimal family engagement with a program may hamper its effectiveness. Family engagement in digital mental health programs is multifaceted and poorly understood, with ill-defined relationships among aspects of participation and program outcomes (ie, what constitutes *effective* engagement). Moreover, little is known about the barriers and facilitators to effectively engaging families at risk for anxiety, who may not be actively seeking treatment.

**Objective:**

*Making Mistakes* is a novel, internet-based, cognitive behavioral preventive program for caregivers and their 5‐ to 7-year-old children, which aims to reduce the risk of anxiety by targeting family transmission of perfectionism or error sensitivity—that is, negative overreactivity to mistakes. This mixed methods study examined multiple facets of parent-and-child engagement in *Making Mistakes*, including adherence, cognitive and affective engagement, barriers, facilitators, and perceived outcomes associated with involvement.

**Methods:**

A total of 87 dyads were included in a quantitative analysis of adherence to the program, including overall adherence and sustained engagement. Eighteen dyads completed qualitative interviews, which were subjected to a qualitative thematic analysis. Finally, a sample of *Making Mistakes* activity journals was qualitatively reviewed and synthesized.

**Results:**

Parent and child adherence were strongly positively correlated (*r*_85_=0.95). Dyads had low adherence to the weekly modules, which did not differ by intervention condition. Completion rates averaged 20%, with adherence declining over time. In contrast, qualitative data indicated high levels of investment in program content and topics, elucidated barriers and facilitators to program engagement, and highlighted numerous psychosocial benefits.

**Conclusions:**

Engagement, operationalized as rote adherence to an intervention developer’s criteria, may insufficiently capture the aspects of participation that are most meaningfully related to positive outcomes. Researchers and intervention developers should prioritize cognitive and affective aspects of engagement with program content, along with promoting and assessing behavioral engagement beyond adherence to program components. *Making Mistakes* shows promise as a low-cost, disseminable strategy to reduce intergenerational risk for anxiety. These findings have particular relevance for digital health programs focused on prevention, parent-child dyads, and anxiety and perfectionism.

## Introduction

### Engagement With Digital Health Programs

Despite the potential of digital health programs (DHPs) to prevent and treat mental health conditions in young children, DHPs have yet to make a significant mental health impact at scale. Many DHPs have faced suboptimal engagement [1], which also tends to decrease over time (ie, “the law of attrition”) [2]. Engagement in DHPs is multifaceted, encompassing affective (eg, enjoyment), cognitive (eg, attention), and behavioral (eg, adherence to intervention prompts) components [3]. Moreover, interaction with a DHP’s user interface is distinct from the enactment of the DHP’s specific behavior change techniques, which is further distinguished from engagement in relevant health behavior changes [4]. Importantly, *effective* engagement is defined as the quantity and timing of energy investment into a stimulus or task that is required to bring about a desired outcome [3]. Understanding effective engagement across multiple dimensions is important to DHP development, dissemination, and evaluation.

Typical patterns of participation vary widely across DHPs, consumers, target conditions, and settings, and depend on how engagement is operationalized [5]. Thus, rather than assuming a rigid, “one-size-fits-all” approach, authors have called for research that focuses on the degree of improvement as a primary metric of engagement success while also exploring multifaceted patterns of involvement [5,6]. Meta-analytic work has shown that engagement in a DHP for mental health is positively associated with clinical improvement, regardless of the intervention focus or whether the intervention is guided or unguided [7]. In one anxiety-focused DHP, the *Unwinding Anxiety* mobile app, completing a higher proportion of modules, as well as specific behavior change techniques, was associated with greater improvements in mental health symptoms [8]. Few preventive, parent-mediated digital interventions for youth 7 years or younger have been rigorously evaluated [9]. Moreover, typical patterns of participation in these programs remain unstudied. Inclusion of consumer perspectives in intervention design, development, and dissemination may help to overcome challenges with program engagement, thereby enhancing outcomes [10]. Understanding consumer engagement in digital mental health interventions is critical, particularly for programs focused on young children.

In parent-mediated interventions, caregiver participation engagement (CPE) refers to active participation in a program, including follow-through with homework and behavior change plans [11]. A meta-analysis found that CPE in youth-focused prevention programs was associated with increases in positive parenting behaviors and decreases in child internalizing and externalizing symptoms [11]. Given that family engagement in mental health intervention programs continues to be a challenge [12], especially in prevention programs [13,14], and that the determinants of CPE are understudied [11], more attention is needed to identify the barriers and facilitators to effective involvement in family-focused behavioral health interventions.

### Families With Elevated Error Sensitivity

In search of empirically informed targets for the prevention of anxiety, over 50 studies to date have linked higher error-related brain activity with anxiety, in both adult and pediatric samples [15-17]. This neural marker, the error-related negativity (ERN), is generated when mistakes are made on a task and can be recorded using an electroencephalogram. A larger ERN in children is related to higher anxiety. Furthermore, an elevated ERN in early childhood independently predicts the risk for clinical anxiety in later childhood [18]. The ERN has been associated with *error sensitivity*, or the tendency to react negatively to one’s own mistakes [19]. Concern over making mistakes is also a hallmark of maladaptive perfectionism [20]. Given these findings, our research team developed an intervention that aims to impact the ERN by targeting overly negative responses to making mistakes (ie, behavioral error sensitivity or perfectionism) in young children [21].

Families high in error sensitivity or perfectionism may face unique challenges in engaging with preventive DHPs. Perfectionistic families may not always seek psychological services; thus, understanding engagement in non–treatment-seeking perfectionistic families is important, consistent with a *prevention* or health-promotion perspective [22]. According to the social expectations model, parents with unreasonably high expectations for their children may have an overcontrolling parenting style, thereby increasing the risk of maladaptive child perfectionism [23]. Moreover, the social learning model (also [23]) suggests that parents pass on perfectionistic behaviors to their children via modeling. These hypotheses are supported by meta-analytic research, which has revealed that parental self-oriented perfectionism, expectations, and criticism positively relate to child perfectionism [24]. Maladaptive perfectionism may result from child traits that dynamically interact with parental factors, such as harsh or critical communication, authoritarian or controlling parenting, psychological control, perfectionistic modeling, and excessive performance feedback focusing on error avoidance [25-31]. Furthermore, behavioral genetics has shown that perfectionism is moderately heritable, meaning perfectionistic families likely share genetic diatheses [20].

Few interventions have targeted perfectionism in youth [32,33] (see [34] for a cursory review). Parent-mediated interventions for perfectionism should target theorized intergenerational mechanisms of children’s maladaptive perfectionism, such as setting excessively high standards, encouraging avoidance of mistakes, and modeling perfectionism [23,24,35]. Published recommendations on preventive interventions for perfectionism include adopting a positive psychology focus; providing a rationale and discussing risk; targeting maladaptive perfectionistic beliefs; providing cognitive reappraisal strategies; promoting a growth mindset, self-acceptance, and self-compassion; and engaging parents in reducing criticism, modeling calm responses to errors, and reiterating anti-perfectionistic messages [36].

### Current Digital Health Intervention Program

In accordance with empirical findings, theory, and guidelines, a preventive DHP targeting error sensitivity in 5‐ to 7-year-old child-parent dyads has shown pilot efficacy [21]. A randomized controlled trial (RCT) of an expanded version of this program is underway.

Mixed methods approaches can elucidate engagement and implementation outcomes in parent-mediated digital interventions (eg, [37-43]). However, little is known about program participation in error-sensitive families. Likewise, there is limited research on family engagement in preventive interventions for anxiety or on DHPs with children under the age of 7 years. Given the increasing importance of DHPs in preventing and treating pediatric mental illness, it is critical to understand *how* families participate in and benefit from these programs, the factors that facilitate participation, and the facets of involvement that contribute most to program success (ie, *effective* engagement). The findings will enhance the potential effectiveness and reach of DHPs by advancing knowledge of typical family participation in preventive DHPs, clarifying the relationship between engagement and program outcomes, and contributing to a deeper understanding of the factors that influence program participation.

In this study, we examine family participation in a preventive DHP targeting error sensitivity in young children and their parents. We delineate patterns of engagement with *Making Mistakes*, including adherence within dyads; perceptions of program outcomes; and cognitive, affective, and behavioral facets of engagement. We also explore facilitators and barriers to family involvement. The research questions are as follows: (1) To what degree do dyads engage in *Making Mistakes* with fidelity, including program adherence and enactment of content learned? (2) How is participation perceived to impact family functioning? (3) What are facilitators and barriers to family engagement? (4) How do qualitative descriptions of engagement contextualize quantitative findings? This study builds on the emerging body of parent-mediated and digital mental health interventions by using mixed methods to explore families’ patterns of program participation. We incorporate robust qualitative methods to contextualize quantitative findings on program adherence and gain an in-depth understanding of participation. Understanding barriers and facilitators to engagement—and what constitutes *effective* engagement—may improve mental health outcomes for families by augmenting the impact of *Making Mistakes* and similar programs.

## Methods

### Recruitment and Participants

This mixed methods study included families who were randomized to the 3 active treatment arms of an ongoing RCT evaluating *Making Mistakes*, a web-based program targeting anxiety risk in 5‐ to 7-year-old children. Parent-child dyads were recruited from a community, nonclinical sample. In the larger study, dyads were interviewed about their experience, including program acceptability and appropriateness. Procedures and methods for the RCT can be found elsewhere ([34]; also see Multimedia Appendix 1).

Parent-child dyads were recruited for quantitative assessment as part of their participation in the larger RCT. Inclusion criteria were as follows: (1) elevated error sensitivity as measured by elevated scores on the Child Error Sensitivity Index, Child Error Sensitivity Index–Parent Report, or the Parent Sensitivity to Child Errors Index [19] and (2) completion of the baseline intervention module. Two dyads were excluded because they did not receive the weekly text message or email reminders as prescribed, due to clerical errors. An additional 7 dyads were excluded for not completing the entirety of the baseline intervention module, resulting in a final sample of 87 dyads for the quantitative assessment (n=30 with both parent *and* child in the active condition [P+/C+], 30 parent-active+child-control [P+/C–], and 27 child-active+parent-control [P–/C+]). Reported reasons for not completing the baseline module indicated barriers related to childcare requirements (ie, siblings), busy schedules, and factors potentially related to child age or developmental level (eg, child refusal to continue the study).

Families were eligible for qualitative interviews if they were randomized into 1 of 3 active intervention arms and completed at least a portion of the baseline module. The lead author (IAM) conducted all interviews, which were solicited across various phases of intervention completion. Random and convenience sampling were used for the first 10 interviewees. Strategic sampling was used for the remainder, which prioritized P+/C+ dyads. Sampling aimed to match or surpass the demographic diversity of the overall participant sample with respect to the timing of the interview vis-à-vis the phase of the study or intervention, race or ethnicity, gender and sexual orientation, and relationship status. Recruitment goals were based on data saturation, which often occurs between 10 and 17 interviews [44]. Data saturation was reached at 18 dyads (n=18 adults, 14 children). Additional information regarding qualitative data collection is reported elsewhere [45].

### Ethical Considerations

Study procedures were approved by Florida State University’s Institutional Review Board (STUDY00000605). Informed consent was obtained from parents, and assent was obtained from children. Participant information was kept private and confidential. We compensated participants with a US $25 gift card.

### Study Design and Procedure

#### Overview

We used a qualitative-dominant, convergent mixed methods design [10,46] to elucidate family engagement in a preventive DHP targeting error sensitivity. The convergent design—wherein qualitative and quantitative data were collected during the same phase of the research process, analyzed separately, and integrated during interpretation—allowed us to gain a deeper understanding of effective family engagement. As a complement to the RCT’s ongoing efficacy evaluation, this study focused on process-oriented features, implementation characteristics, and multidimensional indices of engagement [10]. The first author conducted the semistructured interviews both virtually and face-to-face in the lab setting. Interviews lasted, on average, 48 minutes and were audio-recorded and transcribed.

#### Intervention

*Making Mistakes* (described elsewhere in greater detail) [34] includes independently delivered child and parent modules. Child content included cognitive tools (eg, “mistakes are how we learn”; “mistakes can be funny”), listening to your “mistake buddy,” and standing up to your “mistake bully,” as well as behavioral components such as making mistakes on purpose (exposure) and challenging oneself to try hard things. Parent or caregiver modules similarly focused on exposure, encouraging “challenge-zone” child behavior, emphasizing effort over outcome, providing psychoeducation about error sensitivity, modeling positive reactions to mistakes, and responding positively to child mistakes. Furthermore, parent modules also included general positive parenting tools, such as the use of positive attention and one-on-one, child-led play (special time). Parents and children, respectively, completed the core intervention: a 45-minute baseline module, which was followed by 6 months of less than 5-minute video lessons, thrice monthly. Paper journal activities corresponded with the weekly content, and parents received weekly text and email reminders with links to access modules accompanied by brief messages to reinforce program content (eg, “You can encourage your child to do something challenging, even if they might make mistakes!”). Links were specific to each weekly video, and parents received 2 separate links (one for weekly parent content and one for child content). Participants “logged in” to each respective module by entering their study ID#, which was used to estimate rates of adherence. Thus, if both the parent and child content were accessed for a respective week, 2 separate ID “logins” were recorded.

### Measures

#### Quantitative Measures: Adherence

Adherence was estimated via metadata extracted from the study website, which logged whether each respective module was accessed at any time between baseline and the 6-month follow-up assessment. If a particular module was associated with 2 or more logins for the same participant, only the first login was counted. As both conditions were housed on the same website, rates of condition contamination for the weekly modules were moderate; 1 active-condition parent (P+) accessed a control module, 2 P– parents accessed an active module, and 1 C– child accessed an active module. Given that all these participants had completed the correct initial (primary) module, they were included in the analyses. A summative measure of adherence was computed for parents and children, respectively, and the proportions of module adherence were calculated by dividing the total number of modules completed by 18. Parent and child adherence were then averaged to create a single adherence score. Finally, for sensitivity analyses, a composite variable was created for each of the 18 modules: whether any member of the dyad (including parent, child, or both) had logged into the respective module.

To characterize *sustained adherence*, a more sensitive metric of engagement, an additional variable was computed for parents and children, respectively: the number of *unique weeks* a participant logged in. Parent and child sustained adherence were averaged into a single score.

#### Qualitative Measures: Semistructured Interviews and Journal Entries

An interview guide [34] was developed for this study based on similar guides (eg, [40]). Interviews adhered to existing guidelines on semistructured interview research, including the use of fieldnotes and the incorporation of author positionality [47-50]. To further characterize patterns of CPE and child participation, a sample of paper journal entries was qualitatively reviewed. These included 32 journals photocopied following the baseline module (n=16) or returned at post treatment (n=16), including child (n=16) and parent journals (n=16). Patterns of engagement were reviewed holistically by the lead author (IAM), with a focus on the degree of completion, thoroughness and coherence of responses, common themes, and behavioral commitment language. The findings were integrated with qualitative and quantitative findings on engagement and fidelity.

### Data Analyses

Adherence was reported descriptively for children and adults, including rates of overall and sustained adherence. A Welch’s ANOVA examined differences in adherence by intervention condition. To interpret the qualitative data, a standard thematic analysis informed by the framework method was performed [51,52] using the NVivo software (version 12.7.0; by Lumivero). This study relied on a codebook generated for a prior study, and data preprocessing is described elsewhere in further detail [45]. Summarily, the analysis used a thematic framework [53], data familiarization, iterative development of a coding scheme and coding, double-coding and coauthor consensus building, recoding, development of a thematic matrix, and interpretation of data via the themes. The thematic matrix was developed using a combination of deductive and inductive processes. Rigor and trustworthiness were established through the use of previously published inquiry methods, strategic sampling methods to achieve a diversity of perspectives, triangulation of multiple data types, consultation, integration of author positionality, negative case analysis, and member checking [47,54].

## Results

### Overview

Our quantitative and qualitative results provided complementary insights. Although quantitative patterns revealed relatively low fidelity of adherence, this did not translate to poor engagement with the content. Importantly, barriers to high-fidelity participation were not always barriers to overall program success. Qualitative results highlighted diverse and idiosyncratic barriers and facilitators to engagement and suggested numerous positive outcomes of the program that, in turn, facilitated engagement.

### Quantitative Findings

**Figure 1.**
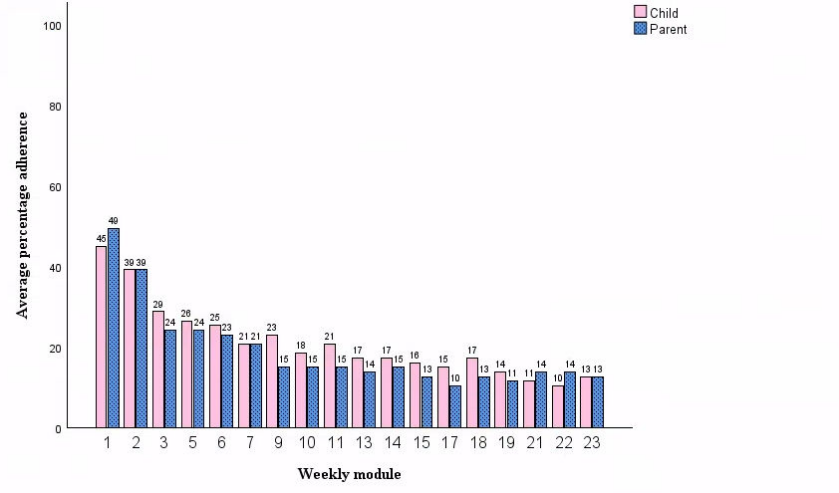
Rates of parent and child adherence by weekly module. “Weekly module” refers to completion of the respective module at any time from baseline to 6-month follow-up.

Demographics are summarized in Table 1; additional demographics of the qualitative sample are reported in Table S1 in Multimedia Appendix 1. Parent and child overall adherence were strongly positively correlated (*r*_85_=0.946, 95% CI 0.919-0.965; *P*<.001). The overall rate of adherence to the weekly modules was approximately 20%, with substantial variability; indeed, 43.7% (n=38) of the parents and 44.8% (n=39) of the children did not log into any of the weekly modules. Parent and child sustained adherence were also strongly positively correlated (*r*_85_=0.949, *P*<.001, 95% CI 0.923-0.966). Table 2 shows adherence by parent and child (see Table S2 in Multimedia Appendix 1 for analogous data within the qualitative sample). Furthermore, adherence declined over time (Figure 1).

**Table 1.** Participant demographics (n=87).

Baseline characteristic	Full sample, n (%)	Interviewees, n (%)
Parent demographics		
Gender		
Woman	79 (90.8)	17 (94.4)
Man	7 (8)	1 (5.6)
Other	1 (1.1)	0 (0)
Education level		
Some high school	1 (1.1)	1 (5.6)
High school diploma or equivalent	4 (4.6)	0 (0)
Some college or 2-year degree	24 (27.6)	5 (27.8)
College degree	29 (33.3)	7 (38.9)
Graduate degree	29 (33.3)	5 (13.9)
Annual family income (US $)		
<10,000	2 (2.3)	1 (5.6)
10,000‐25,000	6 (6.9)	1 (5.6)
25,000‐40,000	10 (11.5)	2 (11.1)
40,000‐75,000	28 (32.3)	9 (50)
>75,000	41 (47.1)	5 (27.8)
Race		
White	64 (73.6)	13 (72.2)
Black	15 (17.2)	3 (16.7)
Asian	1 (1.1)	0 (0)
Other	7 (8)	2 (11.1)
Ethnicity		
Not Hispanic or Latino	81 (93.1)	16 (88.9)
Hispanic or Latino	5 (6.9)	2 (11.1)
Relationship status^a^		
Married or remarried	61 (73.5)	13 (72.2)
Divorced, single	4 (4.8)	2 (11.1)
Single, never married	8 (9.6)	2 (11.1)
Long-term relationship	10 (12)	1 (5.6)
Child demographics		
Gender		
Girl	46 (52.9)	10 (55.6)
Boy	41 (47.1)	8 (44.4)
Race		
White	54 (62.1)	11 (61.1)
Black	15 (17.2)	3 (16.7)
Asian	1 (1.1)	0 (0)
Other	17 (19.5)	4 (22.2)
Ethnicity		
Not Hispanic or Latino	75 (86.2)	14 (83.3)
Hispanic or Latino	12 (13.8)	4 (16.7)

a n=83 for relationship status and parent age (n_missing_=4). Full sample: mean parent age 36.6 (SD 6.0; range 22-52) years; mean child age 6.2 (SD 0.8; range 5-8) years. Interview sample: mean parent age 36.4 (SD 6.3; range 25-48); mean child age 6.2 (SD 0.9; range 5-8).

**Table 2.** Overall adherence rates and sustained adherence to the weekly modules (n=87 dyads)^a^.

Dyad member	Number of unique weeks		Number of total modules		Percentage of total modules	
	Mean (SD)	Median (IQR)	Mean (SD)	Median (IQR)	Mean (SD)	Median (IQR)
Parent	2.3 (3.7)	1 (0-2)	3.4 (5.2)	1 (0-4)	19 (29)	6 (0-22)
Child	2.2 (3.5)	1 (0-3)	3.8 (5.6)	1 (0-6)	21 (31)	6 (0-33)
Average	2.3 (3.5)	0.5 (0-2.5)	3.6 (5.3)	1 (0-6)	20 (30)	6 (0-33)

a Modules were delivered 3 out of 4 weeks, with assessments given every fourth week. Adherence ranged from 0 to 18 for number of modules completed (both parent and child); for unique weeks, it ranged from 0 to 14 (child) and 0 to 15 (parent).

ANOVA assumptions were not met (skewness=1.98; kurtosis=3.15; nonhomogeneity of variance). Thus, a Welch ANOVA, with Games-Howell post hoc comparisons, was used to test for differences in average sustained adherence (ie, number of unique active weeks) by intervention condition (P+/C+: mean 2.37, SD 3.48; P+/C–: mean 2.77, SD 4.26; P–/C+: mean 1.56, SD 2.54). The test was not significant (*F*_2,54.84_=1.05, ω^2^_random=−0.002, 95% CI −0.012 to 0.038; *P*=.36), indicating no differences in sustained adherence rates by condition. Post hoc comparisons revealed that sustained adherence in P+/C– was not significantly greater than in *P*+C+ (95% CI −2.02 to 2.82; *P*=.92, or P–/C+, 95% CI −1.01 to 3.43; *P*=.39); nor was there a difference between P–/C+ and *P*+C+ (95% CI −2.74 to 1.12; *P*=.57). (As a sensitivity analysis, we fit a population-averaged logistic model using generalized estimating equations with exchangeable working correlation to account for within-dyad dependence across weeks. The Condition × Week interaction was not significant (Wald *χ*²_2_=4.36; *P*=.11), indicating that completion trajectories across modules did not differ by condition. This result converged with the primary Welch ANOVA findings.)

### Qualitative Themes

#### Overview

Table 3 shows a summary of qualitative themes and subthemes with example quotes. The themes titled (1) diverse family characteristics and needs and (2) program benefits are described in greater detail below. Patterns of journal completion revealed meaningful parental participation in the baseline intervention module. Journal review revealed detailed, specific written responses indicating deep cognitive and affective engagement with the content, as well as concrete behavioral intentions. A review of completed child journals indicated that some journal activities (eg, intentional misspelling tasks) were inappropriate for some younger children and that adult assistance facilitated journal completion. In addition, children’s journal entries showed a mix of internally consistent responses (eg, endorsed positive self-talk such as “Everyone makes mistakes!” and did *not* endorse “trouble making mistakes”; ie, error sensitivity) and inconsistent responses (eg, circling *all* response options available, even when conflicting).

**Table 3.** Qualitative themes and subthemes with example quotes^a^.

Theme and subtheme	Example quote
Diverse family characteristics and needs	
Broad applicability	“… she kind of fell into all the categories … being shy, being critical of herself … and like what other people thought, um, that fit her like a tee. Um, she has big feelings, deep feelings and... it resonated.” (Parent 15)
Life circumstances and family structure	“I really have a desire to model trying new things, and putting myself out there, like being vulnerable to maybe not being great at first and, you know, working to get better … It’s just so hard with kids and … I have four kids, [aged] seven and under.” (Parent 4)
Program benefits	
Mindful parenting	“I’m, like, trying to be more mindful of it … Because I normally would overreact ... I feel like, ‘Oh, my God, I’m such— like, ‘I can’t believe I did that’. (Parent 9)”
Ripple effects	“I tried to change the way I do things, which, honestly, it positively affects her … she’s a lot more open, willing to talk and, um, you know, she doesn’t get as … upset or anything, or have breakdowns and all.” (Parent 6)
Improved parent-child relationship	“We have completely changed up everything [we do] in the house… If she did something wrong, we would say … ‘is that a good choice?’… ‘what are some better choices?’ … ‘because if you do this, this is what happens’... So now it’s completely different … We redirect, and we talk about it and … actually figure—solve the problem.” (Parent 6)

a Eighteen individual interviews were conducted with parent-child dyads, which were coded and subjected to a thematic analysis.

#### Diverse Family Characteristics and Needs

##### Overview

Families reported diverse barriers and facilitators to engagement. However, interviewees felt that *Making Mistakes* was broadly appropriate for their family and could offer at least *something* to other families. “I think it definitely should be a thing that, that’s like, done by every parent” (Parent 12). Some respondents expressed that the program might be most useful for families of children with behavioral or emotional challenges, or overwhelmed parents in need of encouragement.

Facilitators and barriers to participation emerged with respect to both parent and child traits. Parental facilitators included a willingness to troubleshoot difficulties, along with perseverance in practicing strategies learned, even in the face of perceived failures or setbacks. Individual child differences also served as facilitators and barriers, such as attention span or ability to sit still, rambunctiousness, and tolerance of uncertainty. “My daughter is a planner. She doesn’t like surprises … She’s not great with … change … or transitions, so I kind of have to talk her through … her plan for the day” (Parent 15).

##### Broad Applicability

Children varied in their error sensitivity and other challenges. Some parents described moderate child error sensitivity, with bouts of low frustration tolerance. Others’ children tended to actively approach challenges and had low error sensitivity. Still others described more significant behavioral or emotional challenges or expressed that their child perfectly fit the program’s description of being highly error-sensitive (eg, shy, perfectionistic, avoids challenging tasks, or easily discouraged). Some parents felt their young children had not yet developed the “bad habits” of responding poorly to their own errors. Indeed, *Making Mistakes* content was suggested in some cases to be even more relevant to other children, including older siblings or others outside the home.

*I also try to be mindful of it with my students … My niece and nephew, I’m like, “They need this …” He is going to be the type of kid that’s at MIT, and he’s gonna be like, real stressed out … and ready to like, [jump] off a bridge if something happens.*

[Parent 9]

Interestingly, parents also varied in their own error sensitivity. Some communicated a desire to break cycles of “intergenerational trauma”—or maladaptive cognitions and behavior. Furthermore, some identified immense difficulty resisting the urge to correct their child’s mistakes, with some equating mistakes with *failure*.

I used to not treat [mistakes] correctly … I am [no longer] … pushing him to, to do all the things perfect[ly], I think, you know mainly [*Making Mistakes*] was for me (chuckles)…

[Parent 18]

*… the way I’ve been raised, is like, always, like push, and, you know, achieve, and kind of compared [myself to others] a lot too. So, mistakes are kind of … not looked upon well … I do just see a value in [the weekly activities], umm, for me personally.*

[Parent 3]

Commonly, parents desired to engage their spouse or coparent in the content. “[My spouse is] a Veteran so … for him, it’s separating that mindset of ‘okay, this is not soldiers, this is a child’… he’s still working on that … because he’s very much sometimes in that military mindset” (Parent 6). Facilitators of parent and spousal or coparent participation emerged, including elevated parental error sensitivity, interest in learning about child development, a growth mindset, and favorable views of science; some postulated that younger and more “progressive” parents would find the intervention compelling. Despite heterogeneity in families’ perceived problems, challenges, and circumstances, interviewees resoundingly felt *Making Mistakes* was applicable in some way.

##### Life Circumstances and Family Structure

Engagement was influenced by circumstances including family composition, busyness, employment status, and unpredictable or temporary stressors. However, no simple pattern of participation emerged; indeed, some with minimal circumstantial barriers engaged with low fidelity, and others with myriad external barriers were highly motivated to engage. Thus, engagement was dynamic, complex, and influenced by multiple factors. Specific barriers included single parenthood, excessive childcare demands (especially having multiple young children), busy family schedules, and a desire to avoid overfilling their child’s plate. Busyness decreased adherence to the program.

Temporary circumstances dynamically impacted interest or ability to engage in *Making Mistakes*, including moving or job loss (barriers); resolution of a problem such as bullying, leading to a reduction in child psychosocial problems (decreased motivation to engage); or having an infant child, which led to higher perceived program relevance yet more barriers to actual participation. Overall, family or household structure, employment, and busyness were viewed as barriers, with short-term stressors (eg, moving, newborn care, child bullying) also contributing to the ability and motivation to participate. However, life circumstances, along with perceived child problems, only explained a portion of the variation in program engagement.

### Program Benefits

#### Overview

Dyads consistently expressed that their family had benefited in some way from *Making Mistakes*, which, in turn, facilitated participation fidelity. Cognitive and behavioral change came from the repeated implementation of strategies including reframing mistakes, modeling positive reactions, and responding positively to children’s mistakes. Parents described positive changes in the intervention targets (ie, child error sensitivity) as well as in their own error sensitivity, parent-child communication, and family emotional health. Cognitive changes (eg, increased awareness, modified beliefs about mistakes) were framed as leading to behavioral changes in parents and their children, as well as in other family members or individuals outside of the family. Interviewees also evidenced internalization of program concepts and strategies. For example, some parents struggled to recall specific program-related changes until the strategies were cued, after which they expressed strongly positive feelings toward the content.

*[This program] put a name to [encouraging her to do hard things] and kind of highlighted and reminded me … to suggest those things and … explain that to her … I do remember thinking about those things, and being like, “that is extremely good information …” [Special time] is something I was not doing before, um, and then I kind of forgot that … this program was the one that kind of like, turned that light on.*

[Parent 4]

Children struggled to identify changes; however, some described internalized cognitive strategies, such as listening to their “mistake buddy,” often with helpful scripts. For some, perceived benefits included reduced performance anxiety and perfectionism, increased positive goal-setting and bravery, and improved attentiveness and self-regulation abilities.

*Before, I tried not to make mistakes that mu- a lot, which I, which I did. And now it’s, and now I know that it’s okay to make mistakes. So, so, it’s just fine for me… I messed up and I was like, “it’s okay!”.*

[Child 12]

#### Mindful Parenting

Interviewees gained increased awareness of their impact on their children through a combination of psychoeducation on error sensitivity and being urged to pay more attention to their own behavior and their child’s psyche.

I wondered why my son gets so angry. And ... [now I understand he gets angry when] he make[s] a mistake, or [when I punish him], stuff like that

[Parent 14]

Parents also gained an understanding of their children’s developmental level, as well as naturalistic opportunities to reinforce healthy concepts.

Mindfulness also facilitated resisting the urge to correct or preempt children’s mistakes. This reduction in “autopilot parenting” seemed to stem from increased awareness of parents’ own behavior, improved frustration tolerance, and tolerance for child messiness. “I would say I’ve learned to be a little bit more patienter [sic]” (Parent 10). Mindfulness also led to more developmentally appropriate expectations of their children, which deemphasized perfection and outcomes, instead emphasizing effort and the “big picture.” Respondents learned to pause before responding, when they would recall program concepts and respond accordingly. Interestingly, parents also highlighted a behavioral outcome not explicitly taught: correcting their own negative responses to mistakes.

*I’ll tell … all my kids like, “Mommy made a mistake,” or “Mommy behaved really poorly. I was, uh, I was upset and I, I said a bad word and that was not nice …” Them knowing that I’m not perfect … it gives them the opportunity to not be perfect, too.*

[Parent 4]

#### Ripple Effects

Indirect effects included reciprocal positive influences between child and parent behavior, changes in parents’ own error sensitivity, and reported benefits to siblings or family members. “I think she did definitely gain some self-confidence, um, for sure ... she does really well with the, with the positive reinforcement and praise and … one-on-one time …” (Parent 17). For some dyads, positive family changes were spurred by the child. For instance, parents intentionally reinforced children’s *Making Mistakes* content, and some children reminded their parents and siblings to respond positively to mistakes. In addition, a family noted that their child’s increased emotional vocabulary facilitated their ability to support him appropriately.

Parents learned to be kinder to themselves, which stemmed from modeling positive reactions to their own mistakes and from frequent, small reminders. “It kind of helped me to get my patience back in line and it, you know, kind of let me realize too, that, ‘okay, you’re Mommy, but you’re not perfect; you’re gonna make mistakes, too’” (Parent 10). Positive changes extended to other children and caregivers, as well. Indeed, *Making Mistakes* provided a rationale for parents to encourage coparents to be more mistake-friendly. Most interestingly, program content spread beyond dyads’ immediate family, including the workplace, schools, extended family, and friends.

*I would say something that’s like, “Oh my God, I’m such an idiot, I left this out overnight.” And she’s like, “No, Mom, no negative self-talk” … I’ll say something like, “Do you know what this is?” … she’ll be like, “An overreaction?” … I think she even told my mom, like, “That’s the mistake bully” (laughing).*

[Parent 9]

*… her brother, um, who is autistic and does not cope well with failure, and doing poorly, and making mistakes … she immediately took those ideas home, and does not just say them when she makes mistakes, she’s helping to express them to him as well.*

[Parent 16]

#### Improved Parent-Child Relationship

Interviewees also described improved parent-child functioning. This included more positive communication and quality time, more openness, and fewer negative disciplinary methods, as well as explanations provided to children in slower and simpler language, leading to reduced conflict and frustration. Parents replaced harsh and less effective discipline, such as spanking or yelling, with more effective and gentler methods, such as talking things through and using positive reinforcement.

*We really [reduced his punishments], because in the past we used to say, “you are not going to play with … your Legos,” or “you’re not going to watch TV, you’re not to go to the park.” And, now we’re being more relaxed with, with him [and only removing video-game privileges] … because it was [too much punishment] for him.*

[Parent 18]

These changes led to children more openly discussing their mistakes with parents. “[She’s more open about] how she feels… there not being like this fear cloud that, you know, (chuckles) ‘Mom’s gonna get upset’, or ‘Mom’s gonna shut down’…” (Parent 6).

Families described myriad benefits from their participation. Improvements were noted as receiving helpful tips, which led to more aware and intentional parenting, in turn leading to improved child behavioral and emotional functioning. While respondents generally felt *Making Mistakes* positively impacted their lives, some families described more profound positive results that dramatically improved family functioning and child emotional health.

## Discussion

### Principal Findings

This study examined participation fidelity, family involvement, perceived outcomes, and barriers and facilitators to engagement in a web-based anxiety prevention program in parent-child dyads. Qualitative and quantitative data showed high levels of engagement in the initial module, albeit low levels of DHP adherence over the 6-month intervention period. Barriers and facilitators included competing demands and program credibility, respectively. Perceived benefits emerged as a key facilitator, resulting from engagement spanning cognitive, affective, and behavioral domains.

### Engagement Barriers and Facilitators

Participants completed, on average, one-fifth of the weekly modules, with engagement dropping off over time; however, the modal dyad completed zero weekly modules. Online video and paper journal completion generally coincided, though more families completed the videos, and a few completed journals without the videos. In contrast to the weekly modules, in-lab engagement in the baseline module activities was high. Differences in lab versus home participation align with research demonstrating the utility of human facilitation in promoting DHP usage [55]. DHPs often face low adherence, which may limit therapeutic benefits [56]. Additional work should clarify the relative importance of—and strategies to optimize—different levels of digital engagement, including mechanical program usage, cognitive engagement with program notifications, and behavioral enactment of nondigital tasks resulting from the program [3]. Our findings suggest that this type of DHP may have a strong impact when integrated with human facilitators at the outset, followed by software-delivered engagement strategies tailored to the individual family’s needs. Future research should determine the optimal forms of involvement needed to attain program benefits [57].

Numerous barriers and facilitators to family engagement were identified. Facilitators included programmatic factors, such as teaching specific content and strategies, rationale provision (ie, psychoeducation on error sensitivity), mobile-friendliness and navigability, regular reminders with repeated exposure to the content, participation in the in-lab baseline module, and approaching *Making Mistakes* as a shared family activity. Facilitators also included family characteristics, such as child enthusiasm for learning; perceived relevance to child *or* parent based on program descriptions of traits associated with error sensitivity (eg, shy, sensitive, socially anxious, reactive); belief in the program’s potential and interest in child psychology and parenting improvement; personal negative experiences with high error sensitivity; and buy-in from co-caregivers. Indeed, the broad applicability of *Making Mistakes* appeared to be a key driver of parent participants’ affective engagement, or emotional investment, with the program, which facilitated behavioral change. Moreover, early improvements in family functioning facilitated buy-in and continued engagement. Facilitators overlapped with those found in previous research, including perceived need, appealing content and delivery, openness to prevention and health promotion approaches, individual motivation and interest, and increased insight into health [38,58]. Our findings on facilitators and barriers shed light on potentially important factors influencing CPE within a DHP [11]. Additional strategies, such as personalization and gamification, may enhance overall program adherence and efficacy.

Both program-related and family-related barriers were identified. Regarding program-specific barriers, some families cited issues related to poor customizability of content and delivery, too many steps to access the content, repetitive or overly simple content, intervention dose (ie, weekly) perceived as daunting, and insufficient incentives to complete the weekly activities. Family-level barriers consisted of low perceived relevance due to parent or child traits; multiple competing demands, especially work and childcare; single-parent status; and temporary stressors, such as moving, separation, or child psychosocial stressors. Barriers overlapped with those found in previous research (eg, [38,42]), including childcare demands, employment, low perceived need, difficulty with program navigability, and perceived burdensomeness. Many barriers were potentially related to social determinants of health and the limited sophistication of the program; therefore, just-in-time adaptive interventions could be developed to equitably address error sensitivity in at-risk families [59]. Of note, the sample only included study completers or partial completers, and thus barriers to initial participation were not captured (eg, see [60]). Overall, barriers and facilitators aligned with those predicted by the health belief model and the theory of planned behavior—such as perceived costs, threats, attitudes, and subjective norms—consistent with research demonstrating support for these models in parenting intervention engagement [61]. These findings also add to the growing body of evidence on engagement in youth mental health services [62,63]. However, the development and testing of theories of family engagement in DHPs will be needed to address barriers and optimize facilitators.

### Program Outcomes

Abundant positive changes were highlighted via qualitative interviews. Commonly cited outcomes included direct intervention targets—including parent sensitivity to child errors and child error sensitivity—though broad changes to parenting and parent-child functioning were also described. Changes to beliefs or thinking (eg, reframing mistakes, increased parenting awareness) seemed to reflect evidence of cognitive engagement. These mental shifts were described as preceding behavioral changes. In addition, improvements in child emotional functioning appeared to be driven by changes in parental behavior, although perceived impacts were bidirectional. Participants described additional unanticipated outcomes, including benefits to siblings, other caregivers, friends, family, and community members. Indeed, these descriptions of wide-ranging improvements appeared to reflect a high level of behavioral engagement with *Making Mistakes*, even despite low rates of adherence to the weekly modules.

This apparent discrepancy—that is, abundant perceived positive outcomes and descriptions of strategies enacted, despite low weekly adherence—merits further attention. The findings support distinctions among aspects of behavioral engagement, such as using a DHP’s interface, enacting specific techniques learned, and making broader changes to health behaviors [4]. It is possible that the initial in-lab intervention module was an adequate dose for many participants, and subsequent program interfacing was not necessary for broader enactment of concepts learned. Alternatively, the simple weekly reminder texts and emails may have spurred continued improvements, even for those who did not complete the activities. The findings extend the literature on CPE within a DHP, suggesting that cognitive and affective engagement, as well as more “downstream” indices of behavioral engagement, may be more important than traditional metrics of DHP *adherence*. To better understand what constitutes *effective* CPE [3,11], future research may directly compare different behavioral engagement strategies and explicitly link them to outcomes of interest.

Qualitatively, families also commented on the positive impact of study involvement, including participation in dyadic games during the lab, as well as routine outcomes monitoring via error-sensitivity measures [34]. Future research should clarify the relative importance of such nonspecific program components, including measurement and observation procedures. Indeed, given the known benefits of measurement-based care in youth mental health services [64] as well as the potential for observer effects or reactivity in parent-child interaction research [65], participant reports of program-related changes to functioning should be interpreted cautiously.

Enhancements to program design and structure may help to overcome common program-related barriers to participation. Beyond well-established DHP design recommendations, such as customizable reminders and intervention frequency, gamification, human facilitation, ease of access, and appealing content and delivery (eg, [66-68]), this study suggests several additional strategies that may enhance engagement. Participants responded well to psychoeducation about *error sensitivity*, including its causes and consequences, which increased the perceived relevance of the program. Additionally, DHPs and caregiver-mediated programs may be enhanced through options to engage cocaregivers in the program. Moreover, the findings suggested that offering the initial in-lab module, along with parent-child games, and the opportunity to approach the program as a dyadic activity may have facilitated program engagement. Finally, regular reminders (nudges), including periodic assessments, may have driven family investment even in the absence of repeated doses of the intervention content. These potential strategies should be empirically validated in future research.

This study has multiple strengths, including integrated qualitative and quantitative data; demonstration of qualitative rigor and trustworthiness; inclusion of young children in data collection; multiple novel foci (ie, DHPs for 5 to ‐7-year-olds, error sensitivity, preventive parent-child program development); intervention targets derived from a neural marker of anxiety; multiple operationalizations of adherence; and a critical focus on multiple aspects of engagement, a common pitfall of DHPs and of caregiver-mediated interventions [56].

This study must also be interpreted in light of several limitations. Both intervention conditions were housed on the same website, and thus, a few participants accessed a video from the other condition. Thus, analyses focused on rates of adherence by condition should be interpreted with some caution. In addition, our measures of behavioral engagement were somewhat limited, including overall adherence and sustained engagement. Future research should incorporate more dynamic and multidimensional metrics, such as the amount of time spent with the intervention or implementing strategies learned; explore temporal patterns of program participation; or use ecological momentary assessment methods to capture behavioral, cognitive, and affective engagement over time. In addition, though diverse perspectives were represented among the qualitative interviews, external validity is limited by the sample’s makeup: mostly White, highly educated, multi-parent homes with relatively high household income. Thus, the results may not be fully generalizable to marginalized families facing additional participation barriers. Future studies must examine *Making Mistakes* engagement in diverse samples.

### Conclusion

Preventive programs for young children and families may yield broad-ranging benefits, including transdiagnostic impacts beyond specific intervention targets. Our findings highlight the importance of qualitative assessment in trials of preventive programs and contribute to the sparse literature on prevention programs for young children [69]. The findings also contribute to the broader literature on perfectionism interventions for young people [36], digital health interventions [9,66] including those for preschool anxiety [70,71], and parent-based interventions for young children [72,73].

These findings support further evaluation of a low-intensity preventive intervention, *Making Mistakes,* which shows promise in promoting positive change within error-sensitive families. *Making Mistakes* included a single-session intervention (see [74]) in tandem with brief reminders or nudge interventions [75]. Future iterations of *Making Mistakes* or similar programs could benefit from further tailoring for very young or preliterate children, including the use of verbal recordings instead of written practice assignments. Finally, to meet the diverse needs of families with elevated error sensitivity (see [31]), *modular* DHPs could be developed to prioritize family preferences and accommodate additional areas of concern to the family [76]. Future research should evaluate the long-term impact of *Making Mistakes* and elucidate family engagement in diverse populations. Importantly, family-focused developmental models of perfectionism, including the social expectations, social learning, and anxious rearing models [23,24,31], should guide efforts to develop and implement engaging digital mental health interventions to prevent the development of pediatric anxiety disorders and cultivate resilience in families.

## Supplementary material

10.2196/79898 Multimedia Appendix 1 Supplemental data and access to Making Mistakes program.

## Abbreviations

CPE: caregiver participation engagement
DHP: digital health program
ERN: error-related negativity
RCT: randomized controlled trial
