# Supplementary material for: Family Engagement in a Digital Intervention Targeting Risk for Anxiety in Parent-Child Dyads: Mixed Methods Study
Source: JMIR Pediatr Parent. 2026 Apr 9;9:e79898. doi: 10.2196/79898 (PMC13064961; doi:10.2196/79898)
Supplement: Multimedia Appendix 1 [file pediatrics-v9-e79898-s001.docx]

**Appendix**

**Supplemental Table 1**

*Characteristics of the Qualitative Study Sample*

| ID | Arm | Timing of Interview | Parent Demographics | Child Age/ Gender | Child # Siblings | Adherence (%) | | |
| --- | --- | --- | --- | --- | --- | --- | --- | --- |
|  |  |  |  |  |  | P | C | Avg |
| 1 | P | Post+ | 48/F, White, graduate degree, married | 7/M | 1 | 94 | 94 | 94 |
| 2 | P | Post+ | 43/F, Latina, some college, divorced | 8/F | 1 | 0 | 0 | 0 |
| 3 | P+C | Post | 48/F, White, immigrant, college degree, married | 7/M | 1 | 78 | 78 | 78 |
| 4 | P+C | Post | 39/F, White, graduate degree, married | 6/F | 2 | 0 | 6 | 3 |
| 5 | P+C | Post | 38/F, White, college degree, married | 5/M | 1 | 0 | 0 | 0* |
| 6 | P | Post | 25/F, White, some college, married | 5/F | 3 | 0 | 0 | 0 |
| 7 | C | Post | 41/F, White, some college, married | 7/F | 2 | 0 | 0 | 0 |
| 8 | C | Post+ | 34/F, Black, graduate degree, married | 5/M | 3 | 0 | 17 | 8 |
| 9 | P+C | Post | 32/F, White, college degree, single | 6/M | 0 | 100 | 100 | 100 |
| 10 | P+C | Post | 33/F, Other, high school diploma/GED, single | 7/F | 4 | 0 | 6 | 3** |
| 11 | P+C | Mod. 1 | 29/F, White, college degree, married | 5/F | 2 | 6 | 0 | 3 |
| 12 | P+C | Post | 27/F, White, some college, married | 7/M | 0 | 72 | 100 | 86 |
| 13 | P+C | Post | 37/F, Black, graduate degree, divorced | 6/F | 1† | 33 | 28 | 31* |
| 14 | P+C | Mod. 1 | 38/F, Black, some high school, married | 7/M | 4 | 17 | 17 | 17 |
| 15 | P+C | Peri | 33/F, White, college degree, married | 6/F | 0 | 22 | 17 | 19 |
| 16 | C | Peri | 34/M, White, graduate degree, long-term relationship | 6/F | 1 | 28 | 50 | 39 |
| 17 | P+C | Post– | 37/F, White, some college, married | 5/F | 3 | 0 | 0 | 0 |
| 18 | P+C | Peri | 40/F, Latina, immigrant, college degree, married | 7/M | 1 | 61 | 67 | 64 |

*Note.* Arm = Intervention condition (P+C = parent and child both in experimental group, P = parent-only [child control condition], C = child-only -parent control condition]. Timing of Interview: Mod. 1 = Immediately following completion of the initial, in-lab module; Peri = Midway through 6-month intervention period. Post = During Final Lab Visit or <30 days Posttreatment; Post+ = 30-75 days Posttreatment; Post– = Following 6-month intervention period, though dyad never completed posttreatment assessment/lab visit; F = Female, M = Male.

*Initial in-lab module only partially completed. **Initial in-lab module only partially completed in-lab; unable to ascertain whether module was later fully completed remotely.

†Parent reported this child has 1 sibling in home and 5 siblings unknown to her.

**Supplemental Table 2**

*Overall Adherence Rates and Sustained Adherence to the Weekly Modules*

|  | # Unique Weeks | | # Total Modules | | % Total Modules | |
| --- | --- | --- | --- | --- | --- | --- |
|  | *M* (*SD*) | Mdn | *M* (*SD*) | Mdn | *M*% (*SD*) | Mdn% |
| Parent | 2.7 (4.3) | 0.5 | 4.8 (6.6) | 0.5 | 27 (37) | 3 |
| Child | 2.9 (4.1) | 1 | 5.4 (7.1) | 1 | 30 (39) | 6 |
| Average | 2.8 (4.1) | 0.5 | 5.1 (6.8) | 0.5 | 28 (38) | 3 |

Note. *n* = 18 (interviewee subsample). This table mirrors Table 2 of the main manuscript.

**Accessing the Making Mistakes Intervention Program**

RCT study website: <https://makingmistakes1.weebly.com/> (use “999” for ID)

Current website housing MM intervention program: <https://anxietyclinicsantacruz.weebly.com/free-perfectionism-intervention.html>
